# Improved Angiogenesis in Response to Localized Delivery of Macrophage-Recruiting Molecules

**DOI:** 10.1371/journal.pone.0131643

**Published:** 2015-07-01

**Authors:** Chih-Wei Hsu, Ross A. Poché, Jennifer E. Saik, Saniya Ali, Shang Wang, Nejla Yosef, Gisele A. Calderon, Larry Scott, Tegy J. Vadakkan, Irina V. Larina, Jennifer L. West, Mary E. Dickinson

**Affiliations:** 1 Graduate Program in Integrative Molecular and Biomedical Sciences, Baylor College of Medicine, Houston, Texas, United States of America; 2 Department of Molecular Physiology and Biophysics, Baylor College of Medicine, Houston, Texas, United States of America; 3 Cardiovascular Research Institute, Baylor College of Medicine, Houston, Texas, United States of America; 4 Department of Bioengineering, Rice University, Houston, Texas, United States of America; 5 Department of Biomedical Engineering, Duke University, Durham, North Carolina, United States of America; 1Biomaterials for Regenerative Therapies Group, Institute for Bioengineering of Catalonia, Baldiri Reixac 15-21, Barcelona 08028, Spain, 2Technical University of Catalonia, Av. Diagonal 647, Barcelona 08028, Spain, 3CIBER-BBN, María de Luna 11, Zaragoza 50, SPAIN

## Abstract

Successful engineering of complex organs requires improved methods to promote rapid and stable vascularization of artificial tissue scaffolds. Toward this goal, tissue engineering strategies utilize the release of pro-angiogenic growth factors, alone or in combination, from biomaterials to induce angiogenesis. In this study we have used intravital microscopy to define key, dynamic cellular changes induced by the release of pro-angiogenic factors from polyethylene glycol diacrylate hydrogels transplanted *in vivo*. Our data show robust macrophage recruitment when the potent and synergistic angiogenic factors, PDGFBB and FGF2 were used as compared with VEGF alone and intravital imaging suggested roles for macrophages in endothelial tip cell migration and anastomosis, as well as pericyte-like behavior. Further data from in vivo experiments show that delivery of CSF1 with VEGF can dramatically improve the poor angiogenic response seen with VEGF alone. These studies show that incorporating macrophage-recruiting factors into the design of pro-angiogenic biomaterial scaffolds is a key strategy likely to be necessary for stable vascularization and survival of implanted artificial tissues.

## Introduction

Most recent clinical successes in tissue replacement are restricted to avascular (cartilage and cornea) [1,2] and thin tissues (skin and bladder) [3,4] that can rely on simple diffusion prior to vascularization post-transplantation. However, the engineering of large, complex organs will not only require methods to maintain normal, *ex vivo* tissue physiology before transplantation, but also a means to promote effective integration of the engineered tissue into the host environment. Therefore, a major goal in the field of tissue engineering is the development of methods to rapidly establish functional and stable vascular networks within artificial tissue matrices. Several strategies have been implemented to address this issue, and it is clear that there is much work to do to both pattern vessels within the engineered tissue as well as to improve the host angiogenic response leading to anastomosis and tissue perfusion [5,6].

An essential first step in the vascularization of transplanted artificial tissues is the induction of a rapid host angiogenic response in which new blood vessels form from pre-existing vessels [7]. One widely investigated method to trigger artificial tissue-induced angiogenesis is to incorporate soluble pro-angiogenic signaling molecules into the tissue scaffold to induce a rapid angiogenic response. Many soluble signaling molecules have been shown to have pro-angiogenic properties such as vascular endothelial growth factor (VEGF), angiopoietin, transforming growth factor (TGF), fibroblast growth factor (FGF), hepatocyte growth factor (HGF) and platelet-derived growth factor (PDGF) and numerous groups have incorporated these factors into a variety different biomaterials for therapeutic use [8]. VEGF and FGF2 are among the most widely used in a variety of natural and synthetic biomaterials [9–13]. However, universal strategies have not yet emerged [8]. For instance, data from some therapeutic trials show tremendous promise using VEGF [14,15], but others indicate that delivery of VEGF via diffusion may not be the most effective way to induce stable vessels either to repair damaged tissues or to promote host perfusion of tissue constructs [16–18], and animal studies show that uncontrolled VEGF activity can stimulate unregulated vessel proliferation, resulting in hemangiomas [19,20]. Thus, researchers have also focused on methods of sustained release of VEGF or crosslinking VEGF to the scaffolds to improve the angiogenic response [11,21,22]. In addition to VEGF, several other factors, alone and in combination, have been shown to have potent pro-angiogenic activity and have been used successfully to promote stable angiogenesis and arteriogenesis, and improve damaged circulation [23–28]. An interesting series of studies by Cao and colleagues have shown that PDGFBB combined with FGF2 had a synergistic effect inducing stable vasculature, whereas VEGF alone resulted in leaky and disorganized vessels [23,29]. Data showing increased pericyte investment suggested that PDGFBB may influence vessel stability by directly recruiting perictytes, but PDGFBB did not have the same synergistic angiogenic effect with VEGF when delivered simultaneously [23,24] arguing that the synergies between PDGFBB and FGF2 may relate to factors other than pericyte recruitment.

In addition to a potential role for pericytes, previous studies have revealed that, both PDGFBB and FGF2 can act as chemotactic factors for macrophages [30–33] which are known to act in a pro-angiogenic manner, supporting angiogenesis and arteriogenesis [34,35]. Specifically, macrophages have been shown to be a source of mitogens and angiogenic factors [35,36] and some studies have suggested that they proceed ahead of sprouting vessels and form tunnels to guide neovascularization *in vivo* [37,38]. Other studies indicate that macrophages can physically interact with endothelial cells and bridge between tip cells to increase anastomosis during development [39,40]. Taken together, these data lead us to hypothesize that the synergistic effect of PDGFBB and FGF2 may depend on pro-angiogenic macrophages that promote robust angiogenesis and neovessel stabilization.

With the goal of improving neoangiogenesis in response to artificial tissue scaffolds *in vivo*, we have used static and vital microscopy to characterize the temporal and spatial cell-cell interactions and angiogenic events that are elicited in response to angiogenic growth factors released from transplanted polyethylene glycol diacrylate (PEGDA) hydrogels [11,41]. Specifically, the cornea micropocket assay and transgenic fluorescent reporter mice were used to investigate the cellular basis for the synergistic effect of PDGFBB/FGF2-releasing PEGDA hydrogels on corneal neo-vascularization. Previous studies have shown that while VEGF fails to promote stable angiogenesis in the cornea, the combination of PDGFBB/FGF2 can promote stable angiogenesis that persists even after the removal of the pellet releasing the growth factors, although the mechanisms that underlie these differences remain unclear [23]. To test whether PDGFBB/FGF2 synergism is related to the recruitment of pericytes and/or macrophages we imaged *NG2-DsRed* and *Csf1r-EGFP* reporter lines, respectively. We found that the combination of PDGFBB/FGF2 activates the infiltration of macrophages several days before robust vessel ingression and pericyte investment. Live imaging of the implanted hydrogels showed a rapid infiltration of macrophages that migrated toward the PDGFBB/FGF2-releasing hydrogels as well as many dynamic interactions between macrophages and ingressing blood vessels. The pro-angiogenic role of macrophages was further confirmed by demonstrating that macrophages derived from bone marrow cells are capable of enhancing endothelial cells cord formation when co-cultured in 3D collagen gels *in vitro*. Finally, by generating hydrogels that simultaneously release VEGF and macrophage colony-stimulating factor (CSF1), we demonstrated that CSF1-mediated macrophage recruitment is sufficient to improve the angiogenic response to VEGF. While CSF1 alone did not elicit an angiogenic response, CSF1 increased macrophage recruitment to the cornea and, combined with VEGF, yielded a robust and stable angiogenic response comparable to PDGFBB/FGF2 induced vessels. These studies show that combined approaches to regulate the host immune response as well as angiogenesis are likely to be needed for successful anastomosis and perfusion of engineered tissues.

## Materials and Methods

### Mice

Homozygous transgenic *Flk1-myr*::*mCherry* and *Csf1r-EGFP* mice were generated as described previously [42–44]. *NG2-DsRed* mice were purchased from The Jackson Laboratory. This study was carried out in strict accordance with the recommendations in the Guide for the Care and Use of Laboratory Animals of the National Institutes of Health. All animal research was conducted according to protocols approved by the Institutional Animal Care and Use Committee (IACUC) of Baylor College of Medicine (assurance number AN-4593).

### Synthesis and purification of 10 kDa PEGDA and PEG-RGDS

The synthesis of 10 kDa acryloyl-PEG-acryloyl (PEGDA) and acryloyl-PEG-RGDS (PEG-RGDS) was described previously [45]. In brief, 10 kDa PEG was dissolved in anhydrous dichloromethane at a concentration of 0.1 mM with 0.4 mM acryloyl chloride and 0.2 mM triethylamine and stirred under argon for 18 hours. PEGDA was then recovered by precipitating in cold ether, filtered and dried in vacuum. The acrylated product was characterized by ^1^H-NMR (Advance 400, Bruker) and store under argon at -20°C until use. PEG-RGDS was prepared by conjugating the cell adhesive ligand Arg-Gly-Asp-Ser (American Peptide) with dry acryloyl-PEG-succinimidyl carboxymethyl (SCM; 3,400 Da; Laysan Bio) at a molar ratio of 1.1:1 in dimethyl sulfoxide. The conjugated product was dialyzed, lyophilized, and stored under argon at -20°C. PEG-RGDS was characterized by using a gel permeation chromatography (GPC) system with a PLgel column (5μm, 500Å) and an evaporative light scattering (ELS) detector (Polymer Laboratories). All reagents were obtained from Sigma-Aldrich unless otherwise noted.

### Synthesis of hydrogel discs for implantation into the mouse cornea

The synthesis of PEGDA hydrogel containing soluble growth factor for implanting into mouse cornea micropocket was described previously [41]. Briefly, hydrogels containing soluble growth factors were crosslinked using a stock solution of the photoinititator 2,2-dimethoxy-2-phenylacetophenone (acetophenone) dissolved in N-vinylpyrrolidone (NVP) at the concentration of 300 mg/ml. This was immediately followed by implantation into the mouse cornea. Specifically, the pre-polymer solution was prepared to contain a 10% polymer weight percentage (100 mg/ml) of PEGDA, 3.5 mM PEG-RGDS, 10 μl/ml acetophenone stock solution, and soluble growth factors. The concentration of human VEGF-165, mouse PDGFBB, and mouse CSF1 were adjusted to 2.67 μg/ul, and mouse FGF2 was adjusted to 0.67 μg/ul in the pre-polymer solution. All the growth factors were purchased from Prospec. 0.12 μl of the pre-polymer solution was injected in between two pre-cleaned, sigmacoted glass slides spaced by a 0.005 inch thick poly(tetra fluoroethylene) spacer and secured with binder clips to make an implant of appropriate size and shape for the cornea. The gels were exposed to UV light (B-200SP UV lamp, UVP, 365 nm, 10 mW/cm^2^) for two minutes to allow crosslinking and immediately implanted into the mouse cornea micropocket.

### Growth factor release from PEGDA hydrogel

To define the release kinetics of the PEGDA hydrogels we used for the *in vivo* cornea micropocket angiogenesis assays, we prepared identical gels with 2.67 μg/ul of VEGF, PDGFBB, and FGF2 in the pre-polymer solutions and the gels were polymerized under UV for 2 minutes then immediately incubated in PBS at 37°C. PBS was collected at hour 0, 1, 2, 3, 4, 5, 6, 24, 72, 168, and 240 to measure the amount of growth factor released from the hydrogel discs. The amount of growth factor in the collected solution was quantified by using the NanoOrange Protein Quantification Assay (Life Technology). Fluorescence was measured on the Infinite 200 PRO plate reader (Tecan) using 485 nm excitation and 590 nm emission wavelengths. Bovine serum albumin (BSA) was used as standard to calculate the amount of proteins released into the solutions.

### Cornea micropocket angiogenic assay

The cornea micropockets were prepared as described by Poché et al [41]. Briefly, mice were anesthetized by intraperitoneal injection of 250 mg/kg 2,2,2-tribromoethanol (Avertin, Sigma-Aldrich). The eye was then topically anesthetized with one drop of 0.5% proparacaine (Bausch & Lomb). To create a micropocket, the eye was proptosed with dull forceps and a 30° microknife was used to make a partial-thickness incision in the mid-cornea approximately 1 mm—1.2 mm from the inferior limbus. A Von Graefe knife was inserted under the edge of the inferior lip of the incision and extended into the corneal stroma creating a micropocket that extends toward the inferior limbus. Hydrogels containing soluble growth factors were then placed on the eye and inserted into the micropocket for later evaluation of the angiogenic responses.

### Cornea flat mount and immunofluorescence staining

Eyes were enucleated and fixed in 4% paraformaldehyde (PFA) at 4°C overnight after mice were euthanized with CO_2_. After two 1X PBS washes, corneas were dissected apart from the rest of the eye and separated from the lens for direct flatmount or immunofluorescence staining. For cornea flatmount, cornea was cut into four quadrants, laid flat on a glass slide, and mounted with Fluormount-G (SouthernBiotech) with the epithelium layer facing upwards. Samples for immunofluorescence staining were fixed again in cold 100% acetone for 30 minutes followed by three 1X PBS washes. Corneas were incubated in blocking buffer (1X PBS with 0.8% Triton X and 2% donkey serum) at 4°C overnight. Macrophage marker rat anti-mouse F4/80 antibody (Life Technologies) were diluted in blocking buffer at the ratio of 1:200 and incubated with corneas at 4°C overnight. After washed with PBS three times, Alexa Flour donkey anti-rat 647 (Life Technologies) was diluted in blocking buffer at the ratio of 1:400 and incubated with corneas at 4°C overnight. Corneas were then washed six times in PBS, 30 minutes each to remove unlabeled secondary antibody. Samples were then incubated with DAPI for 20 minutes and mounted flat with Fluormount-G

### Quantitative analysis of vessel density/lacunarity and phagocyte density

For vessel and phagocyte density quantification, mounted corneas were examined using a Plan Apochromat 20X/0.75 NA objective on the Zeiss LSM 510 META confocal microscope. A Multitime macro (Zeiss) was used to collect 2 X 2 tiled z-stack images with a step-size at 5 μm to cover the region of implanted hydrogel and the full thickness of the cornea. The density of vessels or phagocytes was defined as the ratio of the number of mCherry or EGFP positive pixels to the total number of pixels in the region twice the radius of the implanted hydrogel throughout the z-stack images. To determine the total length of vessels, the open snake tracing algorithm in FARSIGHT software to skeletonize the vessel structures in 3D [46] and the total length throughout the z-stack images was determined and normalized to the volume occupied by the implanted gels. For measuring lacunarity, z-stack images were acquired on the surface of the hydrogel by a C-Apochromat 40X/1.2 NA water immersion objective with a step-size at 2.5 μm on LSM 510 META. Phagocyte density and lacunarity measurements were calculated with custom MATLAB (Mathworks) programs as described previously [47].

### Speckle variance optical coherence tomography (SV-OCT) for comparing the tissue perfusion of induced vessels in mouse cornea

To compare the differences of tissue perfusion induced by PDGFBB/FGF2 and VEGF, CD1 mice implanted with hydrogels were anesthetized with Avertin and positioned on the stage of a home-built spectral-domain optical coherence tomography (OCT) system described in [48] with a central wavelength of ~810 nm (bandwidth of ~110 nm). To capture the variance of the speckle intensity, ten frames are taken from each scanning position within the three-dimensional volume. For one position, the speckle variance (SV) signal is obtained through calculating the variance values between every two frames and selecting the maximum values for each pixel of the frame. Thus, imaging contrast is created between the stable cornea and the induced vessels that have the circulating blood cells [49]. After generating the SV images for all the spatial locations of OCT frames, three-dimensional data set is obtained and rendered as volumetric representation using Imaris software. Vessel density is quantified by dividing the volume of the vessel SV signal by the volume of the cornea OCT signal (Bitplane).

### Vital imaging of phagocyte dynamics and phagocyte/endothelial cell interactions

*Csf1r-EGFP* ^*+/tg*^ *; Flk1-myr*::*mCherry* ^*+/tg*^ mice implanted with hydrogels were anesthetized with Avertin and positioned on a Zeiss LSM 5 LIVE confocal microscope. For analyzing phagocyte dynamics, mice were imaged at 3 hours post-implantation. For monitoring phagocyte/endothelial cell interactions, mice were imaged after day 4 post-implantation. The eye was oriented and immersed in lubricant gel drops (GenTeal, Alcon) on top of a No. 1 cover glass. Time-lapse images were taken by using a EC Plan-Neofluar 10X/0.3 NA objective every 30 seconds for 30 minutes or every 90 seconds for 5 hours for phagocyte dynamics analysis. From the time-lapse images, cell density was determined by using the analyze particle function in ImageJ (NIH). Imaris (Bitplane) and custom programs developed in MATLAB were used for phagocyte migration displacement and migration velocity quantifications.

### Total RNA extraction from corneas and qrtPCR

A total of six corneas were pooled together for each condition and digested with 1 mg/ml type II collagenase in 1X PBS for 2 hours at 37°C. After digestion, the solution was pipetted rigorously to completely homogenize cornea tissues. Homogenized corneas were then spun down for total RNA extracted by using TRIzol reagent. Extracted RNA was then cleaned with the RNeasy Mini Kit (Qiagen) following manufacturer’s instructions. cDNA was synthesized by using the Superscript III first strand synthesis kit with Oligo(dT)_20_ and random hexamer priming (Life Technologies). qrtPCRs were performed by using TaqMan qrtPCR primers and Universal qrtPCR Master Primer Mix on a StepOnePlus Real-Time PCR System (Life Technology). The following TaqMan primers were used in this study: *Csf1r* (Mm01266652_m1), *Gsr* (Mm00439154_m1), and *Emr1* (Mm00802529_m1). qrtPCR was performed under the following conditions: 50°C for 2 minutes, 95°C for 2 minutes, 40 cycles of 95°C for 15 seconds and 60°C for 30 seconds. To determine the relative quantification of gene expression, qrtPCR was performed at 250 ng cDNA in five replicates among corneas implanted with blank gel, VEGF-, and PDGFBB/FGF2-releasing gels. GAPDH expression was used for normalization. For data analysis, the Pfaffl method was used to determine relative gene expression ratios [50].

### Cell culture and generation of bone marrow-derived macrophages

Human umbilical vein endothelial cells (HUVECs, Lonza) were maintained at 37°C in a 5% CO_2_ environment in EGM complete medium (Lonza) that contained bovine brain extract (BBE), hydrocortisone, ascorbic acid, human epidermal growth factor (hEGF), GA-1000 (gentamicin, amphotericin-B), heparin, and 2% fetal bovine serum (FBS). HUVECs were used between passages 4 and 8 for experiments performed in this study. To generate bone marrow-derived macrophages (BMDMs), *Csf1r-EGFP* ^*+/tg*^ mice were used for bone marrow extraction as described [51]. Briefly, *Csf1r-EGFP* ^*+/tg*^ mice were euthanized and tibias from both legs were removed. Both ends of the tibias were cut off and bone marrow cells were flushed out with sterile 1X PBS into a petri dish using a 27 gauges needle attached to a 1 ml syringe. Bone marrow cells were than transferred into 15 ml conical tubes and resuspended in 10 ml of BMDM culture medium (1X DMEM high glucose medium supplemented with 20% fetal bovine serum, 100 μg/ml streptomycin and 100 U/ml penicillin, Life Technology). Cell suspensions were then homogenized by gentle pipetting and passed through a 45 μm cell strainer to remove debris. Bone marrow cells were washed twice with the culture medium and then seeded at the density of 5 X 10^6^ cells/plate on 10 cm non-tissue culture treated petri dishes. Cells were supplemented with 40 ng/ml CSF1 and incubated in a humidified incubator at 37°C and 5% CO_2_ to differentiate bone marrow cells to macrophages. For all experiments, differentiated BMDMs were used between passages 2 to 4.

### HUVECs and BMDMs co-culture in three-dimension (3D) collagen gels

For HUVECs and BMDMs encapsulation, collagen pre-gel solution was prepared at 2.5 mg/ml as described [52]. HUVECs were encapsulated in the collagen gel at the density of 2 X 10^6^ cells/ml with 4 X 10^5^ cells/ml BMDMs. Collagen gels with 2 X 10^6^ cells/ml HUVECs were used as control. Ten microliter of collagen pre-gel solution with cells were added to each well of the ibidi u-Slide Angiogenesis and incubated at 37°C in a 5% CO_2_ environment for 30 minutes for the collagen gels to solidify. EGM complete medium supplemented with 40 ng/ml VEGF, 40 ng/ml FGF2 and 40 ng/ml CSF1 were added to each well and incubated in a humidified incubator at 37°C with 5% CO_2_ for 24 and 72 hours. Collagen gels were fixed in 4% paraformaldehyde for 1 hour followed by three 1X PBS washes. Samples were then incubated in blocking buffer (1X PBS with 0.8% Triton X and 2% goat serum) at room temperature for 4 hours. Mouse PECAM (R&D) was diluted at the ratio of 1:200 in blocking buffer and incubated with collagen gel at 4°C overnight. After washed with 1X PBS three times, Alexa Fluor goat anti-mouse 546 (Life Technologies) was diluted in blocking buffer at the ratio of 1:400 along with DAPI (1:500) and incubated with samples at room temperature for 2 hours. Samples were washed three times in 1X PBS and mounted with Fluormount-G. Samples were than examined by Zeiss LSM 780 confocal microscope (Carl Zeiss). Z-stack images were taken using an EC Plan-Neofluar 10X/0.30 NA objective at 0.7 zoom with a step-size of 15.81 um. Tube number and tube length were quantified in 3D using the open snake tracing system with FARSIGHT software [46].

### Statistical analysis

All experiments were performed with n ≥ 3. Student’s t-test, one-way ANOVA, and Kruskal-Wallis test were performed to compare values using the SPSS (version 18.0, IBM) and Prism (version 6.0) with significance set at *P* < 0.05.

## Results

### Vessels induced by PDGFBB/FGF2-releasing PEGDA hydrogels exhibit greater density and structural organization compared to VEGF-induced vessels

**Fig 1:**
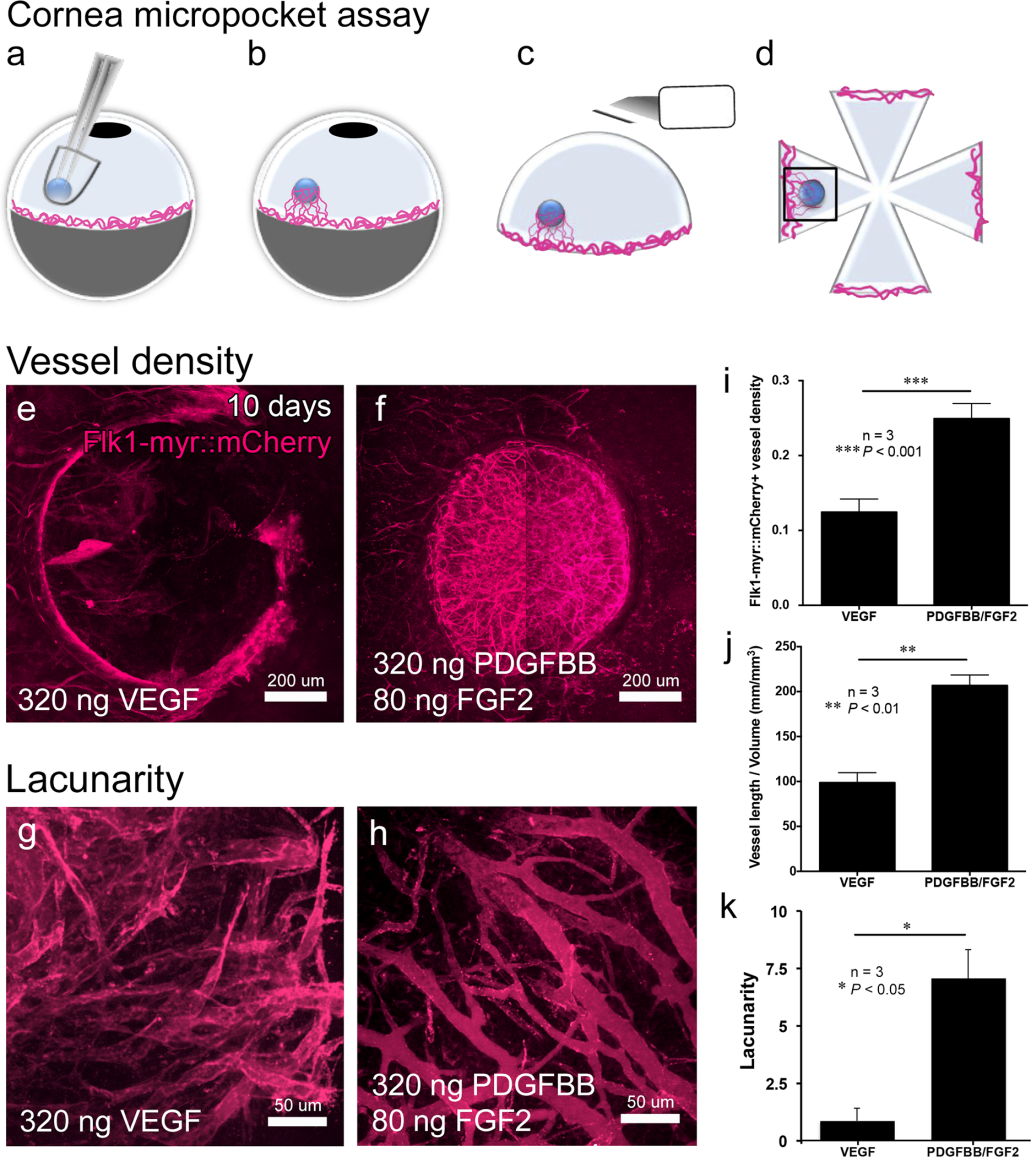
The combination of PDGFBB and FGF2 induces robust angiogenesis with well-organized vascular structure. A schematic diagram of the cornea micropocket assay (a-d). A hydrogel with releasable pro-angiogenic factor is implanted into the cornea micropocket (a) and induced vessels grow toward implanted hydrogel from the limbal region (b). For analysis, the cornea is dissected from the rest of the eye (c) and flat mounted as four quadrants (d). Flk1-myr::mCherry+ vessels induced by VEGF (e) or PDGFBB/FGF2 (f) were examined at 10 days post-implantation. Vessel density induced by PDGFBB/FGF2 was significantly higher than VEGF (i, n = 3, ***P<0.001), as well as the total vessel length (j, n = 3, **P<0.01). Vessel structure was compared between VEGF- (g) and PDGFBB/FGF2- (h) induced vessels in regions that have similar vessel coverage on the surface of hydrogel. VEGF–induced vessels appeared as disorganized with extensive sprouting while PDGFBB/FGF2-induced vessels appeared more established and lumenized. Lacunarity parameter measurements indicated that PDGFBB/FGF2-induced vessels have larger spaces between vessels compared to VEGF-induce vessels (k, n = 3, *P<0.05). Values are presented as mean ± SEM.

For the studies reported in this paper, we have used a modified version of the classical corneal micropocket assay [11,41] combined with transgenic reporter mice to define strategies to optimize neoangiogenic responses elicited by PEGDA hydrogel scaffolds. Previous studies have indicated that PDGFBB/FGF2-induced neoangiogenesis leads to greater vessel stability than VEGF and we sought to understand the cellular basis for this observation [23]. Briefly, hydrogel discs were encapsulated with either 320 ng VEGF or a combination of 320 ng PDGFBB and 80 ng of FGF2 and immediately implanted into the corneal stroma of *Flk1-myr*::*mCherry* ^+/tg^ transgenic mice [42]. This transgenic line brightly labels endothelial cell membranes with the mCherry fluorescent reporter and provides a means to track changes in vessel morphology [43]. After 10 days post-implantation, corneal flat mounts were generated and the induced vasculature was imaged with confocal microscopy (Fig 1A–1D). Consistent with previous reports [23,27,28], PDGFBB/FGF2-releasing hydrogels induced a more robust angiogenic response toward the implanted hydrogel as well as greater vessel coverage of the hydrogel (Fig 1E and 1F). Quantification showed that the combination of PDGFBB/FGF2 induced vessels with a significantly higher density than VEGF alone (Fig 1I, *P*<0.001). The total length of vessel branches also revealed a significant increase in vascularization induced by PDGFBB/FGF2 when compared to VEGF (Fig 1J, *P*<0.01). Higher magnification images of the induced corneal vasculature also suggested a distinct difference in vascular morphology between the VEGF and PDGFBB/FGF2 groups. The VEGF-induced vasculature appeared to be qualitatively less organized and have more sprouts than the PDGFBB/FGF2-induced vessels, which appeared more continuous and lumenized (Fig 1G and 1H). To quantify these dramatic structural differences, we performed measurements of the lacunarity parameter to define the space-filling properties of the induced vessel beds [47]. Here, corneal regions that had similar vessel density on the surface of the hydrogels were used for our quantification and we found that the PDGFBB/FGF2-induced vasculature exhibited a significantly higher lacunarity as compared to the VEGF-induced vessels (Fig 1K, *P*<0.05). Overall, these findings demonstrate that PDGFBB/FGF2-releasing hydrogels not only induce robust angiogenesis, but the induced vessels also form a more organized microvasculature structure than those induced by VEGF.

To confirm that the differences in vascularization induced by PDGFBB/FGF2 vs. VEGF was not related to different release kinetics of the growth factors from the gels, we directly determined the time-course of growth factor release from VEGF, FGF2, and PDGFBB gels *in vitro*. The data indicate that within the first 6 hours, VEGF release was initially slower but by 6 hours and throughout the rest of the 10 day period, VEGF, FGF2, and PDGFBB have statistically indistinguishable release patterns (S1 Fig). Thus, there is no difference in growth factor availability during the time when vascularization is being established in the cornea between days 3 and 10, supporting the idea that different growth factors may elicit different host responses.

### Corneal tissues surrounding the implanted PDGFBB/FGF2-releasing hydrogels were better perfused than corneas containing VEGF hydrogels

**Fig 2:**
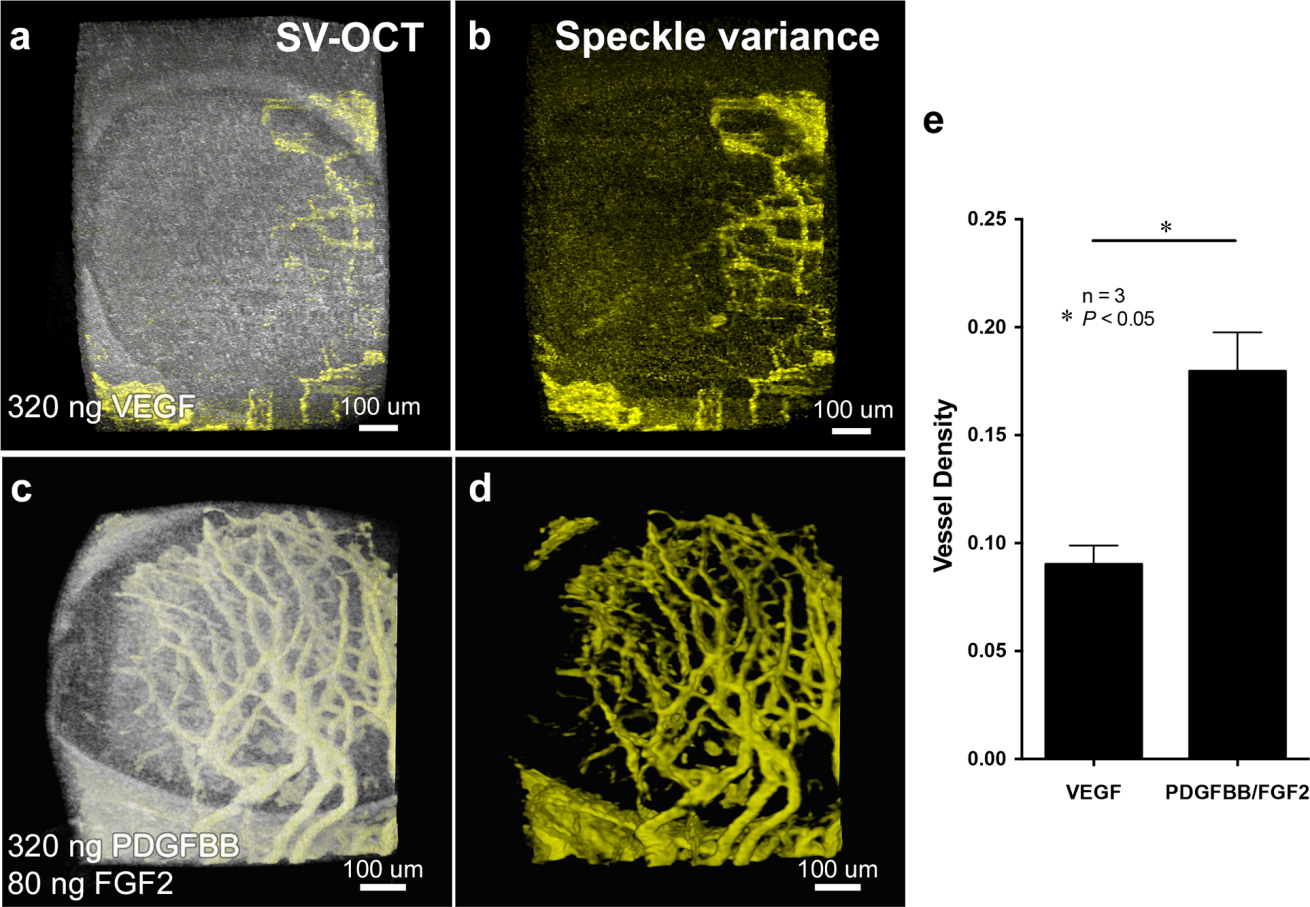
Corneal tissues surrounding the implanted PDGFBB/FGF2-releasing hydrogels were better perfused than corneas containing VEGF hydrogels. 3D reconstructed SV-OCT images of the mouse corneas implanted with VEGF (a) and PDGFBB/FGF2 (c) hydrogel for 10 days. SV-OCT analysis showed a greater number of perfused vessels in the corneas implanted with PDGFBB/FGF2-releasing (d) hydrogels when compared to corneas implanted with VEGF-releasing hydrogels (b). Vessel density measurement by calculating the ratio of the volume of SV signal to the tissue volume from the OCT signal showed that PDGFBB/FGF2 induced higher density of perfused vessel than VEGF in the corneal tissues (e, n = 3, *P<0.05).

In addition to the measurements of vascularization provided above, we also used speckle variance optical coherence tomography (SV-OCT) imaging to determine differences in vessel perfusion between VEGF and PDGFBB/FGF2 induced vessels in the mouse corneas. While Doppler imaging is traditionally used to quantify blood flow using OCT, Doppler measurements are angle-dependent and the signal is only detected from the velocity component parallel to the scan beam; thus, Doppler can be difficult to perform in microvascular beds that are flat and perpendicular to the image plane as in our experiments in the cornea. However, SV-OCT is an excellent, well-established [53–55] angle-independent method to assess perfusion in such cases. The movement of blood as it flows through even small vessels induces a high variation in the speckle intensity obtained when using OCT imaging. These variations can be computed to produce image contrast, providing an image map of perfused vessels in 3D without using any external contrast agent [53,55]. Low or absent SV signals signify poor or absent blood flow, whereas positive pixels in SV-OCT images represent significant variations of the speckle intensities, indicating perfusion of the vessels [49]. CD1 mouse corneas implanted with either 320 ng VEGF or a combination of 320 ng PDGFBB and 80 ng of FGF2 for 10 days were imaged using OCT and analyzed for speckle variance (SV-OCT). Fig 2 shows the 3D reconstructed SV-OCT images of the mouse corneas implanted with VEGF (Fig 2A and 2B) and PDGFBB/FGF2 hydrogels (Fig 2C and 2D and S1 Movie). Consistent with our previous data showing increased vessel density, we also detected a significantly greater number of perfused vessels in the corneas implanted with PDGFBB/FGF2-releasing hydrogels when compared to corneas implanted with VEGF-releasing hydrogels (Fig 2E, *P<*0.05). These findings suggest that PDGFBB/FGF2 not only induces a more robust angiogenic response with more organized vessel structures, the induced vessels are also better perfused than those induced by VEGF.

### PDGFBB/FGF2 signaling increases the number of Csf1r-EGFP+ cells but not NG2-DsRed+ pericytes recruited to growth factor releasing hydrogels

**Fig 3:**
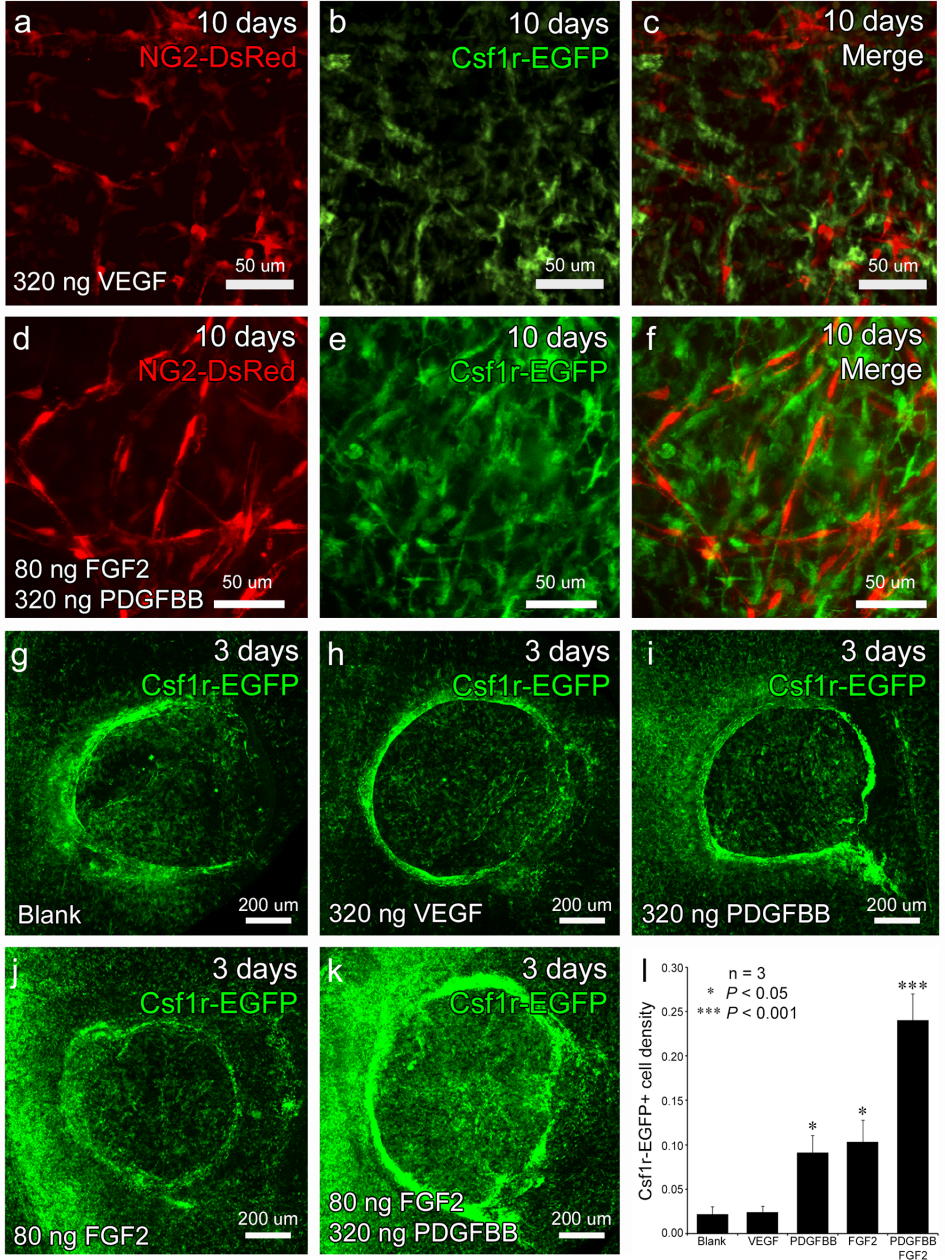
An increased number of Csf1r-EGFP+ cells appear in the cornea ahead of sprouting vessels and pericyte recruitment. At 10 days post-implantation, both VEGF- (a) and PDGFBB/FGF2- induced (d) vessel beds exhibit clear NG2-DsRed+ pericyte investment, but the pericytes in VEGF sample appeared less elongated indicating a possible difference in vascular coverage. There were qualitatively fewer Csf1r-EGFP+ cells in the VEGF (b) samples as compared to PDGFBB/FGF2 (e), which also showed more structural heterogeneity. DsRed+ EGFP+ double positive cells were never observed (c and f). Csf1r-EGFP+ cells were examined at 3 days post-implantation. Compared to blank (g), VEGF- (h), PDGFBB- (i) and FGF2-releasing gels (j), the PDGFBB/FGF2-releasing gels (k) exhibited a greater density of Csf1r-EGFP+ cells in the region occupied by the hydrogels (l, n = 3, * *P*<0.05, *** *P*<0.001).

The apparent differences in PDGFBB/FGF2-induced vessel density, structural organization, and tissue perfusion led us to test the hypothesis that PDGFBB/FGF2 signaling results in the recruitment of perivascular support cells which confer these properties. To test this hypothesis, we implanted VEGF (320 ng) and PDGFBB/FGF2 (320 ng/80 ng) releasing hydrogels into the corneas of *NG2-DsRed* ^*+/tg*^ *; Csf1r-EGFP* ^*+/tg*^ transgenic mice which allow us to simultaneously track pericytes and phagocytes [44,56]. Corneal flat mounts were analyzed at 10 days post-implantation when the corneal microvascular structure has been established and remodeled on the surface of the hydrogels [41,43]. At this time, there is a clear investment of NG2-DsRed+ pericytes within both VEGF- and PDGFBB/FGF2-induced vasculatures on the surface of the hydrogels (Fig 3A and 3D). However, in the case of the VEGF-induced vessels, the pericyte morphology appeared less elongated and vessel coverage less uniform as compared to PDGFBB/FGF2. When examining Csf1r-EGFP+ cells, we observed that the PDGFBB/FGF2 hydrogel implanted corneas exhibited a dramatic increase in EGFP+ cells as compared to VEGF (Fig 3B and 3E). Furthermore, the Csf1r-EGFP+ cells in the PDGFBB/FGF2 group had more diverse cellular morphologies with some being elongated and lining vessels while others exhibited more branching and localization to intervascular spaces (Fig 3C and 3F). At no point did we observe DsRed+ EGFP+ double positive cells, suggesting that the pericytes are not derived from the Csf1r-EGFP+ cells.

To confirm the increased density of Csf1r-EGFP+ cells is correlated with the PDGFBB/FGF2 angiogenic synergism, we compared the Csf1r-EGFP+ cell density in corneas induced with hydrogels delivering different pro-angiogenic factors. Hydrogels containing releasable VEGF (320 ng), PDGFBB (320 ng), FGF2 (80 ng), and PDGFBB/FGF2 (320 ng/80 ng) were implanted in the corneas of *Csf1r-EGFP* ^*+/tg*^ mice and corneal flat mounts were analyzed 3 days later by confocal microscopy. Blank PEGDA hydrogels with no growth factors were used as control. Projected z-stack confocal images showed that the corneas implanted with PDGFBB/FGF2-releasing hydrogels showed the greatest presence of Csf1r-EGFP+ cells compared to the rest of the group (Fig 3G–3K). We next quantified Csf1r-EGFP+ cell density by determining the ratio of the number of EGFP+ pixels to the total number of pixels covered by the region within twice the radius of the implanted hydrogel (Fig 3L). These data showed no significant difference between the blank (0.02 ± 0.01) and VEGF-releasing hydrogels (0.03 ± 0.01). However, we did detect a significant increase in EGFP+ cell density when we compared the PDGFBB (0.10 ± 0.02) and FGF2 (0.09 ± 0.02) releasing hydrogels to the blank and VEGF groups. Moreover, corneas treated with PDGFBB/FGF2-releasing hydrogels exhibited the greatest increase in Csf1r-EGFP+ cell density being more than twice as dense as PDGFBB or FGF2 alone (0.22 ± 0.03, *P*<0.001). This dramatic increase in Csf1r-EGFP+ cell density correlates well with the previously identified angiogenic synergism exhibited by PDGFBB and FGF2 [23] and further support the possibility that phagocytes contribute to PDGFBB/FGF2-mediated angiogenesis.

### PDGFBB/FGF2-responding Csf1r-EGFP+ cells are comprised of different macrophage subtypes

**Fig 4:**
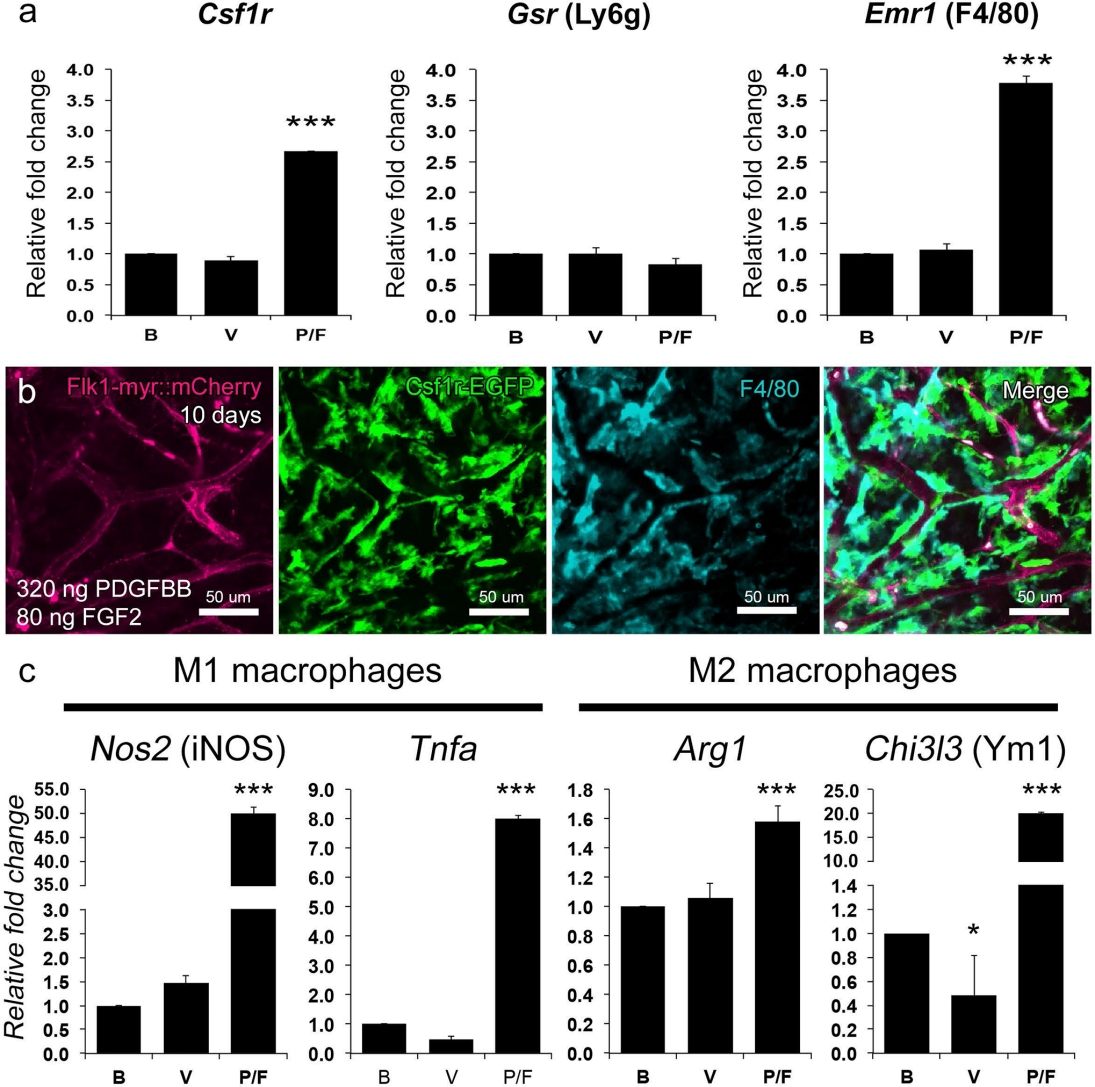
PDGFBB/FGF2-responding Csf1r-EGFP+ cells are comprised of different macrophage subtypes. qrtPCR analysis was performed on corneas implanted with blank, VEGF-, and PDGFBB/FGF2-releasing hydrogels 5 days post-implantation (a). The phagocytes marker *Csf1r* was significantly upregulated in PDGFBB/FGF2-implanted corneas reflecting the increase in Csf1r-GFP+ cells. The neutrophil marker *Gsr* (Ly6g) showed no difference among all three groups whereas *Emr1* (F4/80) expression was significantly up-regulated in the PDGFBB/FGF2-implanted corneas indicating an enrichment of mature macrophages. F4/80 immunofluorescence and co-localization with Csf1r-EGFP, at 10 days post-implantation, further confirmed the presence of macrophages among the Flk1-myr::mCherry+ vessels within PDGFBB/FGF2-implanted corneas (b). qrtPCR indicated that both M1 (*Nos2* and *Tnfa*) and M2 (*Arg1* and *Chi3l3*) macrophage marker genes were significantly up regulated in the PDGFBB/FGF2 implanted corneas compared to blank or VEGF hydrogels (c). Abbreviations: Blank (B); VEGF (V); PDGFBB/FGF2 (P/F).

To further define the phagocyte subset that infiltrates the cornea preceding and during PDGFBB/FGF2 mediated angiogenesis, we performed gene profiling experiments. Total mRNA was extracted from corneas implanted with blank, VEGF and PDGFBB/FGF2-releasing hydrogels 5 days post-implantation and these samples were subjected to qrtPCR. Consistent with our previous quantification of the Csf1r-EGFP+ cell density, *Csf1r*, which encodes colony stimulation factor 1 receptor, was significantly up-regulated in the PDGFBB/FGF2 corneas (Fig 4A). The neutrophil-expressed *Gsr* (Ly6G) [57] showed no difference among blank, VEGF, and PDGFBB/FGF2. However, *Emr1*, which encodes macrophage-specific membrane glycoprotein F4/80 [58], was significantly up-regulated in the PDGFBB/FGF2 corneas. This apparent increase in macrophage markers was further confirmed by immunofluorescence staining of F4/80 at day 10 (Fig 4B). Most, if not all, of the Csf1r-EGFP+ cells associated with the Flk1-myr::mCherry+ vessels also stained positive for F4/80, indicating that many of these cells are indeed macrophages. qrtPCR was also performed to distinguish which macrophage activation state is more dominant in association with PDGFBB/FGF2 angiogenic synergism. Our result indicates that both classical-activated M1 (*Nos2* and *Tnfa*) and alternative-activated M2 (*Arg1* and *Chi3l3*) marker genes were highly up regulated in the PDGFBB/FGF2 implanted corneas compared to corneas implanted with blank or VEGF hydrogels (Fig 4C), suggesting the presence of both M1 and M2 populations in the cornea implanted with PDGFBB/FGF2.

### PDGFBB/FGF2 signaling results in Csf1r-EGFP+ macrophage recruitment into the cornea via cell migration

**Fig 5:**
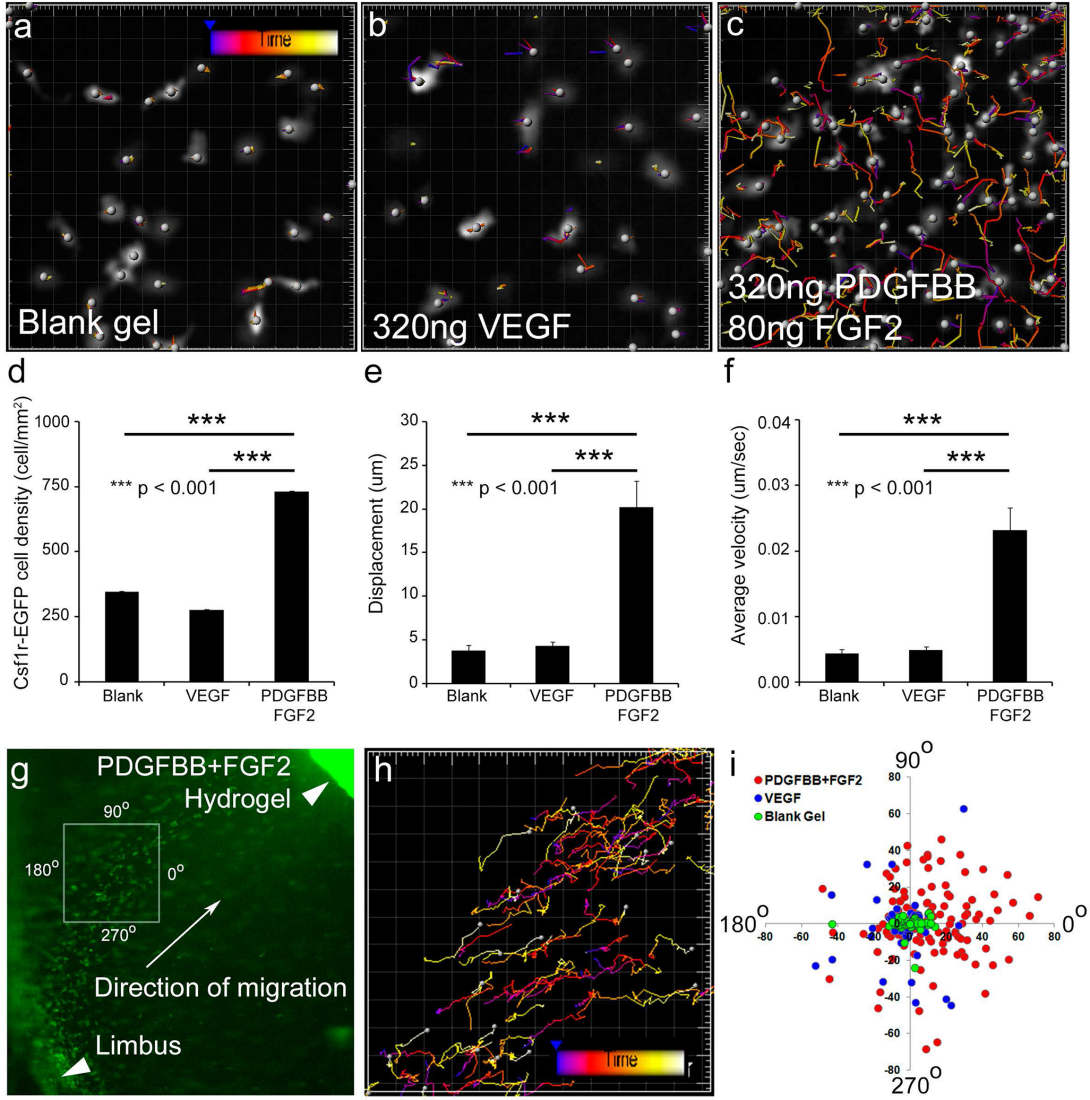
PDGFBB/FGF2-stimulated directional migration of Csf1r-EGFP+ phagocytes. Time-lapse, *in vivo* imaging of *Csf1r-EGFP* ^*+/tg*^ corneas, starting at 3 hour post implantation, showed enhanced Csf1r-EGFP+ cell motility in response to PDGFBB/FGF2-releasing hydrogels as compared to blank or VEGF (a-c). From these movies (3 separate movies from 3 individual mice and each condition), Csf1r-EGFP+ cell density (d), total displacement (e) and migration velocity (f) were calculated and shown to be greater for the PDGFBB/FGF-implanted corneas. Time lapse imaging of directional migration of the Csf1r-EGFP+ phagocytes was performed at 3.5 hour post implantation (g) and analyzed by Imaris (h). The displacement and angle of displacement of each Csf1r-EGFP+ cell was plotted on a rose plot (i) and indicated that the Csf1r-EGFP+ cells stimulated by PDGFBB/FGF2 migrated toward implanted hydrogels.

We next sought to determine whether the observed increase in corneal Csf1r-EGFP+ macrophages, in response to PDGFBB/FGF2-releasing hydrogels, is due to proliferation of resident cells or migration of cells from outside of the cornea. To distinguish between these two possibilities, we performed time-lapse live, confocal imaging of the mouse cornea, which allowed us to directly observe how Csf1r-EGFP+ macrophages respond to PDGFBB/FGF2- and VEGF-releasing hydrogels *in vivo*. Since the PDGFBB/FGF2-mediated increase in corneal Csf1r-EGFP+ macrophages is already obvious by 24 hours post-implantation, we decided to perform vital imaging starting at 3 hours post-implantation so that we might capture the earliest contributing events. We imaged Csf1r-EGFP+ macrophages within hydrogel-implanted corneas every 30 seconds for 30 minutes and then used image analysis software Imaris to track the EGFP+ cell movements over the course of the time lapse. Csf1r-EGFP+ macrophages showed limited migratory behavior when either blank hydrogels or VEGF hydrogels were implanted into the cornea (Fig 5A and 5B and S2 Movie). However, in response to PDGFBB/FGF2, Csf1r-EGFP+ cells appeared to be greater in number and extremely dynamic, with extensive migration (Fig 5C and S2 Movie). Quantitative measurements of cell density, total displacement, and the average velocity confirmed that the number of Csf1r-EGFP+ cells was significantly higher in the PDGFBB/FGF2 induced cornea and that these cells exhibited greater motility with longer displacement and faster migration velocities (Fig 5D–5F, *P*<0.001). It should also be noted that at no point in these movies did we observe Csf1r-EGFP+ macrophages undergoing mitosis. Consistent with this observation, phospho-histone H3 (PH3) immunofluorescence staining of corneal flat mounts at 6 hours post-implantation showed no significant change among blank, VEGF and PDGFBB/FGF2 samples (S2 Fig). Thus, these data suggest that the increase in Csf1r-EGFP+ cells observed in corneas treated with PDGFBB/FGF2 hydrogels is not due to proliferation *in situ*, but is consistent with robust recruitment to the cornea.

Given the dynamic nature of the Csf1r-EGFP+ macrophages, we used live imaging to determine the origins of the migrating cells. Imaging at lower magnification from 3.5 to 5 hours post-hydrogel implantation, at an interval of every 90 seconds, we found Csf1r-EGFP+ macrophages migrating from the limbal vessels toward the implanted PDGFBB/FGF2-releasing hydrogels (Fig 5G, arrow and S3 Movie). Sustained migratory events from the limbal vessels were only rarely observed when blank or VEGF hydrogels were implanted (data not shown), consistent with migration data shown in Fig 5A–5F. Analysis of the migratory tracks of these cells clearly showed a strong orientation from the limbal vessels toward the PDGFBB/FGF2-releasing hydrogel (Fig 5H). To further quantify the differences between Csf1r-EGFP+ macrophages migrating in response to PDGFBB/FGF2 versus VEGF or blank gels, we generated a rose plot of the displacement and angle of displacement for individual Csf1r-EGFP+ macrophages responding to the various conditions (Fig 5I). Csf1r-EGFP+ macrophages exposed to a source of PDGFBB/FGF2 demonstrated a trend of longer displacement and a directional recruitment toward the implanted hydrogel. In total, these data indicate that the increase in Csf1r-EGFP+ macrophages, observed well ahead of angiogenesis in response to PDGFBB/FGF2-releasing hydrogels, is due to directed cell migration into the cornea from the limbus vessels rather than cell proliferation of resident corneal cells.

### Live imaging reveals direct interactions between Csf1r-EGFP+ macrophages and neoangiogenic sprouts

**Fig 6:**
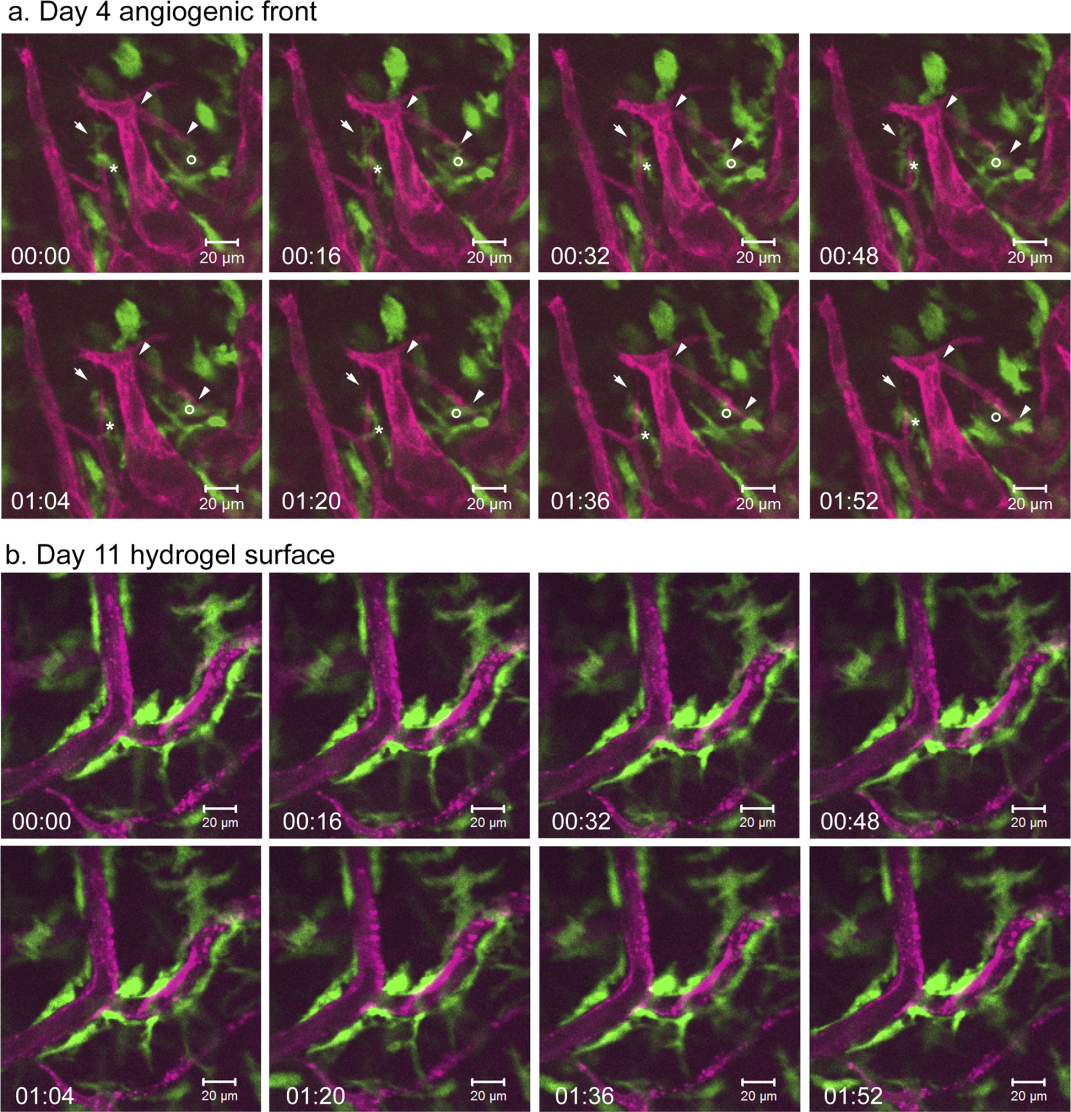
Live imaging of dynamic cell-cell contact between Csf1r-EGFP+ macrophages and Flk1-myr::mCherry+ vessels. Live imaging was performed at the angiogenic front of *Csf1r-EGFP* ^*+/tg*^ *; Flk1-myr*::*mCherry* ^*+/tg*^ corneas implanted with PDGFBB/FGF2-releasing hydrogel at day 4 (S5 Movie) and day 11(S6 Movie) post-implantation. A series of images extracted from time lapse movie indicates that macrophages bridge endothelial tip cells potentially facilitating vessel anastomosis and vessel sprouting at day 4 (a) (see text for details). At 11 days post-implantation, the macrophage population is less dynamic and exhibits an elongated, pericyte-like morphology lining the vessels suggestive of a possible long-term supportive role in maintaining vessel stability (b).

To better understand the role of Csf1r-EGFP+ macrophages during angiogenesis, we performed intravital, time-lapse imaging experiments on *Csf1r-EGFP* ^*+/tg*^ *; Flk1-myr*::*mCherry* ^*+/tg*^ mice 4 days post implantation of PDGFBB/FGF2-releasing hydrogels. Previous data has suggested that macrophages may aid in vessel anastomosis during development [39] and our *in vivo* imaging data also identify interactions between Csf1r-EGFP+ cells and endothelial tip cells that are consistent with this finding. We observed that among the most highly dynamic cells, were those interacting with the tips cells, even bridging between two adjacent tip cells at the angiogenic front (S4 and S5 Movies). Fig 6A shows the extracted sequence from time-lapse movie obtained at the angiogenic front at 4 days post-implantation (S5 Movie). One macrophage (asterisk) behaved as a cellular chaperon by bridging two adjacent sprouts and actively interacting with the filopodia of the endothelial cells. However, the macrophage left its bridging position and migrated down along one of the sprouts. After the macrophage migrated away, immediately the filopodia interaction between the sprouts (arrow) broke apart followed by retraction of the sprouting branch. However, it is still not clear from our *in vivo* imaging data whether macrophages promote anastomosis or prune unnecessary branches or both. In addition to interacting with the fusion point between two sprouts, live imaging also showed that close association of macrophages with sprouting tips could potentially aid in endothelial branch elongation through the extracellular matrix (ECM). In the same extracted sequence (Fig 6A and S5 Movie), another macrophage (circle) was closely associated and actively interacting with the filopodia of the sprouting tip. The macrophage maintained its close association with the filopodia of the sprouting tip as the sprout elongated toward another established vessel. We also observed that Csf1r-EGFP+ macrophages behaved differently among more established, less dynamic vessels. When we examined macrophage-endothelial interaction at day 11 on the surface of implanted PDGFBB/FGF2 hydrogel, Csf1r-EGFP+ macrophages became less dynamic as compared to the subset that was interacting with the angiogenic front (S6 Movie). Instead, these more static macrophages surrounded the vessels in a manner reminiscent of pericyte coverage (Fig 6B) suggesting a possible long-term role as support cells to maintain vessel homeostasis.

### Macrophages promote HUVEC cord formation *in vitro*

**Fig 7:**
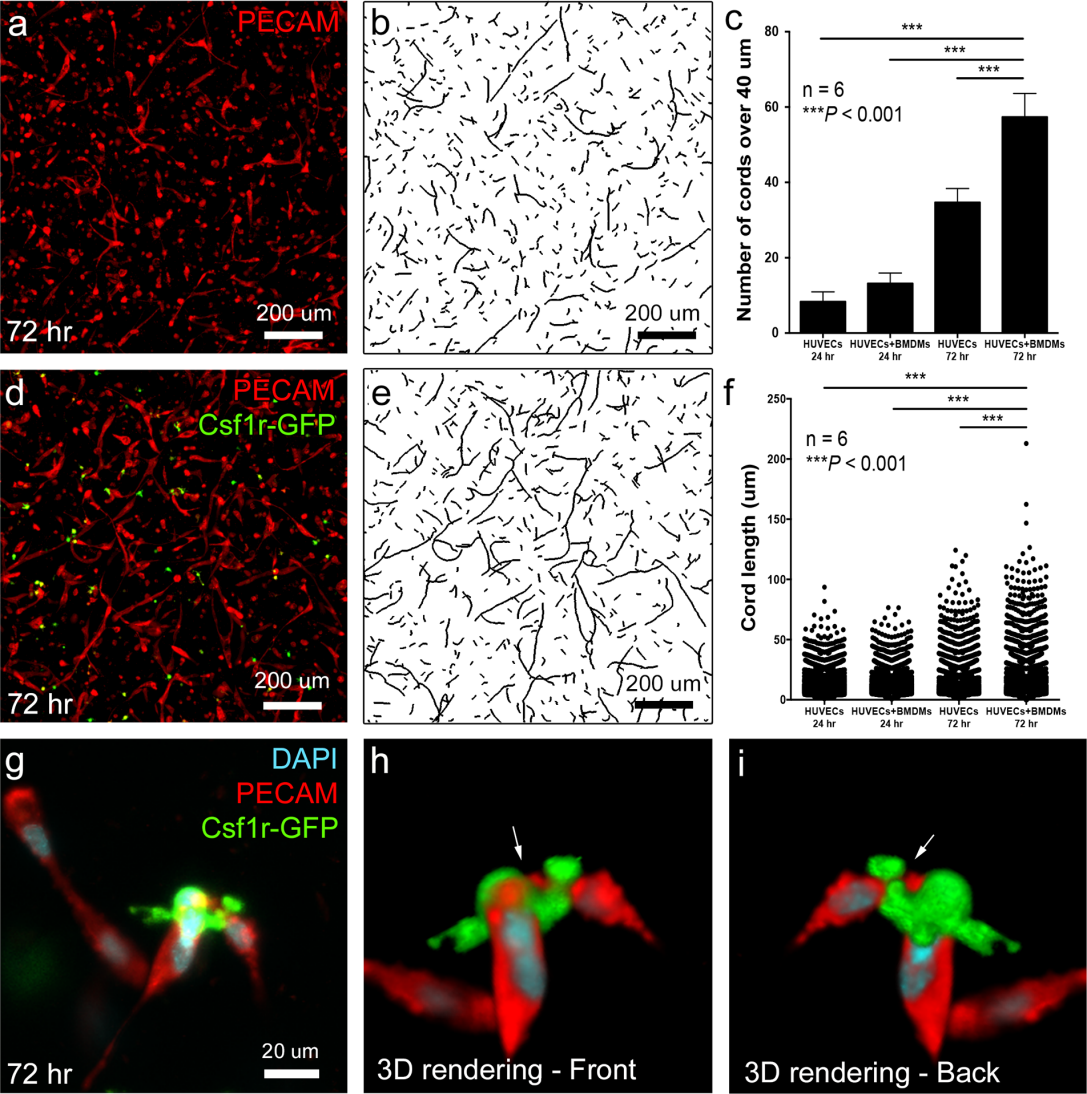
Bone marrow derived macrophages (BMDMs) increase HUVEC cord length and number in 3D collagen gels. After 24 and 72 hours, cord structures formed by HUVECs (a) or HUVECs + BMDMs at 5:1 ratio (d) were fixed and immunofluorescence stained with PECAM, imaged with confocal microscopy and skeletonized by using open snake tracing algorithm in FARSIGHT software (b and e). In the BMDMs/HUVECs co-cultures, the number of cords over 40 um (c, n = 6, ***P<0.001, One-way ANOVA) and the distribution of cord lengths (f, n = 6, ***P<0.001, Kruspal-Wallis) were significantly increased at 72 hours. Panel g shows an example of a macrophage physically associating with HUVECs and 3D rendering of the z-stack at different angles indicated that the macrophage was bridging the junction between two separate cord structures (arrows in h and i).

Our *in vivo* corneal micropocket experiments revealed a pro-angiogenic role for recruited macrophages as contributing to the greater stability and structural organization observed in PDGFBB/FGF2-induced corneal vessels [23,27]. To further corroborate this, we next performed an endothelial cell cord formation assay to determine whether macrophages are sufficient for promoting the formation of vascular structures *in vitro*. Specifically, we encapsulated mouse bone marrow-derived macrophages (BMDMs) from *Csf1r-EGFP* ^*+/tg*^ mice with human umbilical vein endothelial cells (HUVECs) in 3D collagen gels (ratio of 1:5) with the presence of VEGF (40 ng/ml), FGF2 (40 ng/ml), and macrophage colony stimulating factor (CSF1, 40 ng/ml) for 24 and 72 hours followed by examination with confocal microscopy. After 72 hours, we performed PECAM immunofluorescence staining to visualize the HUVECs and found that the HUVEC/BMDM co-culture exhibited an increased number of cord structures as well as longer cord lengths as compared to the HUVEC only culture (compare Fig 7A to 7D). For ease of visualization, we also employed the open snake tracing algorithm in FARSIGHT software to skeletonize the cord structures in 3D (compare Fig 7B to 7E) [46]. Subsequent quantification of the images revealed that both HUVEC-only and HUVEC/BMDM co-cultures induced a similar number of cords at 24 hours with similar length distributions (Fig 7C and 7F). However, by 72 hours, the number of cords longer than 40 μm was significantly increased (Fig 7C, *P*<0.001, One-way ANOVA) in the BMDM/HUVEC co-cultures versus the HUVEC-only cultures. The distribution of cord lengths in the co-cultures was also significantly increased (Fig 7F, *P*<0.001, Kruspal-Wallis). High magnification images of macrophage/endothelial cell co-cultures showed that macrophages become closely associated with endothelial cells, even bridging between adjacent cells, possibly to aid in joining adjacent cords (Fig 7G–7I). The bridging between endothelial cells is highly reminiscent of the interactions between macrophages and tip cells *in vivo*, as revealed in our intravital imaging experiments (see Fig 6A). These *in vitro* data further support a pro-angiogenic role for macrophages during vessel formation, perhaps working to join the ends of nascent vessels, and defines a new strategy for creating vascularized scaffolds *ex vivo*.

### Macrophage recruitment by CSF1 greatly enhances VEGF-induced angiogenesis *in vivo*

**Fig 8:**
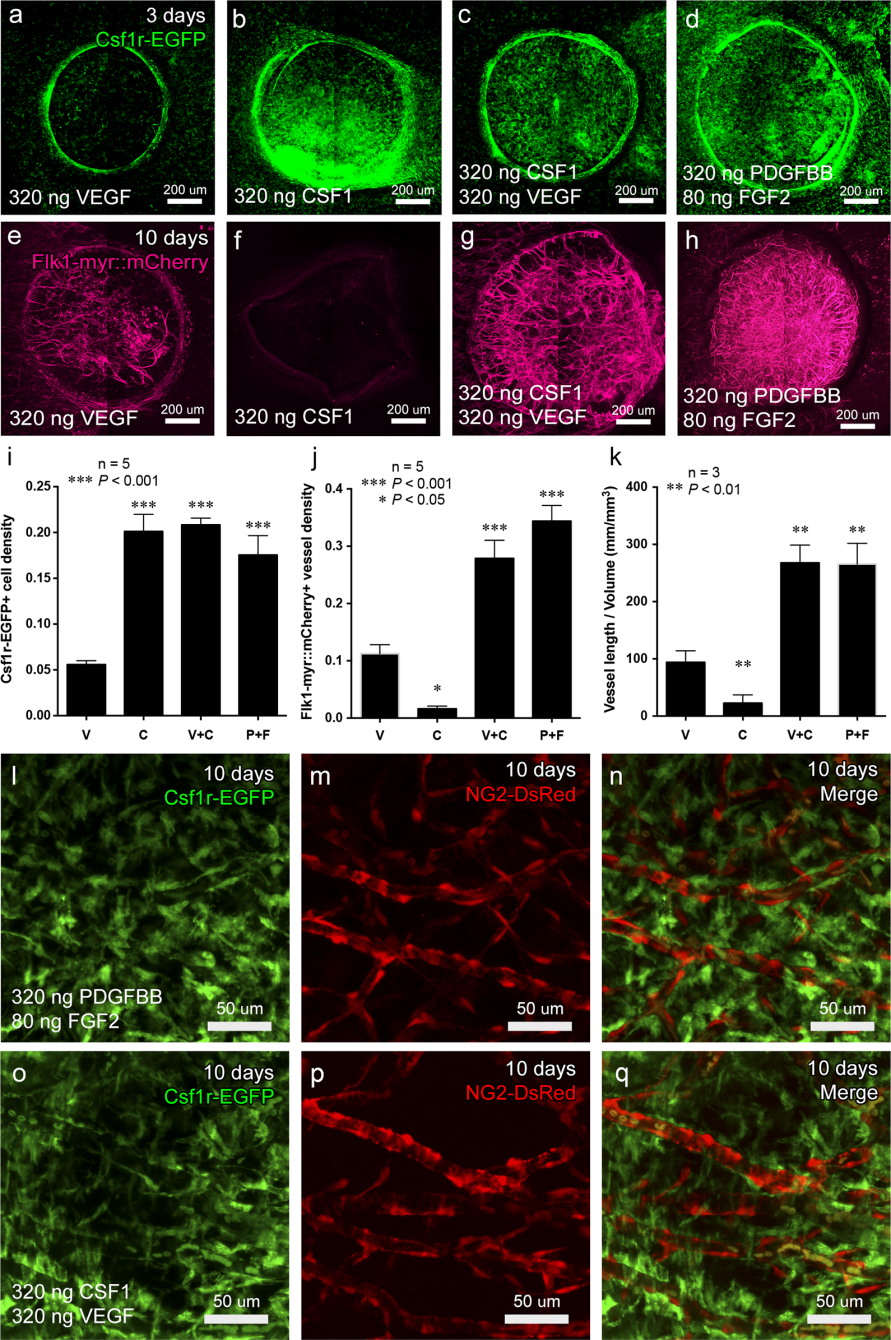
CSF1-mediated macrophage recruitment enhances VEGF-induced angiogenesis. PEGDA hydrogels encapsulated with VEGF (320 ng), CSF1 (320 ng), VEGF (320 ng) + CSF1 (320 ng) or PDGFBB (320 ng) + FGF2 (80 ng) were implanted in *Csf1r-EGFP* ^*+/tg*^ or *Flk1-myr*::*mCherry* ^*+/tg*^ mice. Quantification of confocal images (a-d) indicated that the density of Csf1r-EGFP+ cells recruited by either CSF1 or CSF1/VEGF at day 3 was significantly higher than VEGF alone and similar to the density of PDGDBB/FGF2 (i). When comparing vessel density and total vessel length at day 10 (e-h), CSF1 alone was non-angiogenic. However, the combination of CSF1/VEGF induced a more robust angiogenic response compared to VEGF alone that was comparable to PDGFBB/FGF2 (j and k). When CSF1 was co-delivered with VEGF, the recruited macrophages not only improved the angiogenic response, but also enhanced pericyte investment on VEGF-induced vessels (o-q), similar to PDGFBB/FGF2 induced vessels (l-n). Abbreviations: VEGF (V); CSF1 (C); VEGF+CSF1 (V+C); PDGFBB+FGF2 (P+F).

Since our data pointed to a role for macrophages in promoting the robust, stable and less hyper-branched vasculature generated in response to PDGFBB/FGF2 signaling, we next sought to determine if macrophages are capable of conferring the same characteristics upon VEGF-induced vessels. Therefore, we performed corneal micropocket assays as before, but now included CSF1 simultaneously with VEGF. CSF1 is a well-known macrophage chemotactic, proliferation and differentiation factor [59,60]. Thus, we reasoned that releasable CSF1 might allow us to recruit macrophages to vessels induced by VEGF-releasing hydrogels. PEGDA hydrogels encapsulated with VEGF (320 ng), CSF1 (320 ng), VEGF/CSF1 (320 ng/320 ng), or PDGFBB/FGF2 (320 ng/80 ng) were implanted in *Csf1r-EGFP* ^*+/tg*^ or *Flk1-myr*::*mCherry* ^*+/tg*^ mouse corneas and flat mounts were imaged at 3 and 10 days post-implantation. Images and density measurements of Csf1r-EGFP+ macrophages at 3 days post-implantation showed that CSF1-, CSF1/VEGF-, and PDGFBB/FGF2-releasing hydrogels recruited comparable density of macrophages (Fig 8B–8D, no significant difference), which were approximately 4 fold higher than VEGF (Fig 8A) releasing hydrogels (Fig 8I, *P*<0.001). To determine the angiogenic potential of CSF1-recruited macrophages, vessel density and total vessel length of Flk1-myr::mCherry+ vessels were examined at day 10 post-implantation. The result shows that while the macrophages recruited by CSF1 alone are non-angiogenic, the combination of CSF1 with VEGF induced a more robust angiogenic response compared to VEGF alone and was comparable to PDGFBB/FGF2 (Fig 8E–8H). Quantification of Flk1-myr::mCherry+ vessel density and total vessel length (Fig 8J and 8K) indicated a significant increase in CSF1/VEGF-induced vessels versus VEGF alone, whereas there were no significant difference between CSF1/VEGF- and PDGFBB/FGF2- induced vessel density and total vessel length. CSF1/VEGF-induced vessels also have similar pericyte coverage when compared to PDGFBB/FGF2 at 10 days post implantation (Fig 8L–8Q) with both exhibiting induced vessels that were highly invested by NG2-DsRed+ pericytes in contrast to VEGF-induced vessels, which showed sparse coverage (Fig 3A–3C). These findings demonstrate that although CSF1-recruited macrophages are not angiogenic in the cornea, specific recruitment of macrophages is sufficient to enhance the angiogenic response and pericyte investment of VEGF-induced vasculature *in vivo*.

## Discussion

Currently, one of the greatest challenges in regenerative medicine is to develop efficacious strategies to promote rapid, stable and physiologically appropriate vascularization of artificial tissue matrices. Toward that end, the last decade has seen a tremendous effort in utilizing pro-angiogenic signaling molecules to induce neo-angiogenesis *in vivo*. A number of studies have focused on utilizing VEGF alone, however, multiple-factor cocktails, such as the simultaneous delivery of PDGFBB and FGF2, have also been shown to induce robust angiogenic responses and with greater vessel stability [23,28]. However, factors such as these also have a wide array of cellular targets and pleiotropic effects outside of the endothelium [61–66]. In order to define the mechanisms underlying the previously described angiogenic synergism exhibited by PDGFBB/FGF2 [23], we utilized live imaging and cell-type specific fluorescent protein reporters to define differences in the recruitment of perivascular and pro-angiogenic immune cells, such as macrophages. Consistent with previous reports [23,27], we observed more robust vascularization and vessel perfusion when PDGFBB/FGF2-releasing hydrogel scaffolds were implanted compared to VEGF-releasing hydrogels. Our studies showed a clear chemotactic response of macrophages toward the PDGFBB/FGF2-releasing hydrogels as early as 3 hours post-implantation, indicating that PDGFBB/FGF2-macrophages were rapidly recruited by the growth factors themselves rather than being induced by the neo-vasculature. We also show that PDGFBB/FGF2-releasing hydrogel scaffolds recruit macrophages that precede the vascular front. Such a result could be explained simply if there was a difference in the release kinetics of the factors from the gel, particularly if PDGFBB/FGF2 were released over a longer period of time. However, *in vitro* release kinetics assays showed that VEGF, FGF2 and PDGFBB are all released with similar kinetics (S1 Fig). Thus, we hypothesized that the improved response to PDGFBB/FGF2 was related to the initial macrophage recruitment that preceded the angiogenic front. We tested this hypothesis by testing whether the response to VEGF could be improved by combining VEGF with CSF1, a known cytokine for macrophages. We indeed found that CSF1+VEGF improved vascularization compared to VEGF alone, yielding a response similar to PDGFBB/FGF2. While previous data has focused on the fact that macrophages express VEGF which may underlie their pro-angiogenic properties [67–69], we showed that CSF1-releasing hydrogels were very efficient in recruiting macrophages into the corneas, but did not induce neo-angiogenesis. These data clearly show that the macrophages themselves cannot supplant the role of traditional pro-angiogenic signals but that they may play important roles to modify or augment the effect of pro-angiogenic factors. Thus, multiple growth factors can improve angiogenesis by eliciting responses from different cell types.

This study highlights the significance of the host cellular microenvironment in promoting and supporting artificial tissue construct vascularization and points to a critical role of macrophages in fine-tuning the angiogenic response induced by growth factors such as VEGF. While our data clearly point to a pro-angiogenic role for macrophages, it is important to consider their tremendous heterogeneity in terms of gene expression and cellular function [70,71]. Indeed, our analysis of PDGFBB/FGF2-recruited macrophages showed no evidence of skewing from inflammatory M1 to reparative M2 macrophage types, although it is possible that this reflects a need to identify new molecular markers that identify macrophage populations which promote stable angiogenesis *in vivo* [72]. Also, monitoring of macrophage dynamics and endothelial cell interactions revealed diversity in dynamic behaviors showing that there are either distinct roles for individual macrophages or that individual macrophages can change behavior and/or the way they interact with other cells.

Other studies have also identified diverse roles for macrophages in angiogenesis and vessel patterning. In the developing retina, myeloid cells were recently shown to use non-canonical Wnt signaling to promote Flt1 expression thereby inhibiting VEGF activity to suppress vessel branching and fine tune the patterning of the vascular plexus [73]. However, since soluble Flt1 is actively expressed by the normally avascular cornea, this mechanism is likely not responsible for the PDGFBB/FGF2-mediated vascular response described here [74]. Another report found that retinal macrophages release VEGF-C to activate VEGFR3 in endothelial tip cells, which reinforces Notch signaling to promote the stalk cell phenotype and controlled vascular branching [40]. Macrophages have also been shown in the zebrafish trunk to display what was described as a “chaperone” behavior to promote vascular anastomosis by mediating contact between adjacent endothelial tip cells [39]. Interestingly, we have observed a sub-population of macrophages that directly interact with tip cells using live imaging of the PDGFBB/FGF2-induced vascular front in the mouse cornea, as well as between BMDMs and HUVECs *in vitro*. We also observed macrophages associated with sprouting vessels in transit toward an established vessel, raising the possibility that macrophages aid in endothelial cell migration and branching possibly by remodeling the ECM as described in other cellular contexts [37,38]. Finally, previous studies have shown that PDGFBB/FGF2-induced vessels persist longer than VEGF-induced vessels [23,27], even when the source of growth factor is removed, suggesting that macrophages may influence vessel stability directly. Our live imaging data shows that macrophages at 11 days post-implantation of PDGFBB/FGF2-releasing hydrogels were much less dynamic, lining the vessels in a pericyte-like fashion and this was not observed for the less stable, VEGF-induced vessels. Thus, macrophages may act to stabilize vessels prior to or in addition to pericyte investment. It is also possible that some of the Csf1r-EGFP cells are pericyte progenitors. We did not observe cells positive for both Csf1r-EGFP and the pericyte marker NG2-DsRed, but more rigorous lineage analysis would be needed to draw firm conclusions.

Traditionally, the development of artificial tissue scaffolds has focused on materials that elicit very little, if any, immune response from the host [75]. However, the growing importance of macrophages in angiogenesis warrants re-evaluation of this stance, as some level of immune response may profoundly improve the time-course and stability of host vascularization. Given that macrophages have been shown to have both pro-angiogenic roles as well as opposite roles in supporting vessel regression [76–78], it may be necessary to develop materials to recruit or support specific classes of pro-angiogenic macrophages (as described above). Furthermore, the imaging data presented here also indicate that it will be important for scaffolds to permit extensive macrophage motility and interactions with endothelial cells. In conclusion, this study identifies macrophages as key cellular players contributing to the angiogenic synergism of PDGF and FGF2 and supports further investigation into their utility in promoting the vascularization of transplanted artificial tissue constructs.

## Supporting Information

S1 Fig *In vitro* study of growth factor release kinetics from PEGDA hydrogels. The release kinetics of PDGFBB, FGF2, and VEGF from PEGDA hydrogels (320 ng/gel) was tested *in vitro*. A fast release of the factors from the gels into PBS solution were observed within the first 6 hours, which followed by a slow release of the remaining factor over a period of 10 days. VEGF release was slower (n = 3, *P*<0.05) between hour 1 to 5 when compare to PDGFBB and FGF2. But by 6 hours and throughout the rest of the 10-day period, VEGF, FGF2, and PDGFBB have statistically indistinguishable release patterns.
(TIF)

S2 Fig The increase density of Csf1r-EGFP+ cells is not due to proliferation of resident macrophages. Z-stack image covering the full thickness of the cornea implanted with PDGFBB/FGF2 hydrogel at 6 hours post implantation shows that PH3+ nuclei are located exclusively in the epithelial layer of the cornea and Csf1r-EGFP+/PH3- cells reside within the corneal stroma (a). Corneas implanted with blank (b), VEGF- (c) and PDGFBB/FGF2-releasing hydrogels (d) were stained with PH3 at 6 hours post implantation. Quantification of the mitotic index showed no significant difference among all three groups (e).
(TIF)

S1 Movie Speckle variance-optical coherence tomography (SV-OCT) shows that the corneal tissue is better perfused when implanted with PDGFBB/FGF2 hydrogel than VEGF. Live imaging of CD1 mouse corneas by using SV-OCT detected a significantly greater number of perfused vessels in the corneas implanted with PDGFBB/FGF2-releasing hydrogels (right) when compared to corneas implanted with VEGF-releasing hydrogels (left) at 10-day post-implantation.
(MOV)

S2 Movie Increased Csf1r-EGFP+ macrophage density and dynamics in the corneas implanted with PDGFBB/FGF2-releasing hydrogels. Live imaging of *Csf1r-EGFP* ^*+/tg*^ corneas implanted with PEGDA hydrogels with no growth factor (blank), VEGF (320 ng), and PDGFBB/FGF2 (320 ng/80 ng) starting at 3 hours post implantation. Limited migratory behavior was observed for Csf1r-EGFP+ macrophages in corneas implanted with either blank or VEGF hydrogels. However, in response to PDGFBB/FGF2, Csf1r-EGFP+ macrophages appeared to be greater in number and extremely dynamic, with extensive migration. Time lapse images were taken at a 30 seconds interval starting at 3 hours post-implantation. The time scale is indicated in mm:ss.
(MOV)

S3 Movie Csf1r-EGFP+ macrophages migrate from the limbal vessels toward the implanted PDGFBB/FGF2-releasing hydrogels. Live imaging of *Csf1r-EGFP* ^*+/tg*^ cornea implanted with PDGFBB/FGF2-releasing hydrogel showed recruitment of Csf1r-EGFP+ macrophages, which migrated from the limbal vessels toward the implanted PEGDA hydrogel. Time lapse images were taken at an interval of every 90 seconds from 3.5 to 5 hours post-implantation.
(MOV)

S4 Movie PDGFBB/FGF2 recruited macrophages actively interact with the filopodia of adjacent tip cells at the angiogenic front. Live imaging of *Csf1r-EGFP* ^*+/tg*^ *; Flk1-myr*::*mCherry* ^*+/tg*^ cornea 4 days post implantation of PDGFBB/FGF2-releasing hydrogel showed one macrophage was bridging between two adjacent sprouting branches and actively interacting with the endothelial cell filopodia at the angiogenic front. Time lapse images were taken at an interval of every 60 seconds over a period of 1 hour and 10 minutes. The time scale is indicated in hh:mm:ss.
(MOV)

S5 Movie Macrophages bridge endothelial tip cells potentially facilitating vessel anastomosis and vessel sprouting. Live imaging was performed at the angiogenic front of *Csf1r-EGFP* ^*+/tg*^ *; Flk1-myr*::*mCherry* ^*+/tg*^ cornea implanted with PDGFBB/FGF2-releasing hydrogel at day 4 post-implantation. One macrophage behaved as a cellular chaperon by bridging two adjacent sprouts and actively interacting with the filopodia of the endothelial cells at the angiogenic front. However, the macrophage left its bridging position (at 00:55:00) and migrated down along one of the sprouts. After the macrophage migrated away, immediately the filopodia interaction between the sprouts broke apart followed by retraction of the sprouting branch. In the same movie, another macrophage was closely associated and actively interacting with the filopodia of the sprouting tip. The macrophage maintained its close association with the filopodia of the sprouting tip as the sprout elongated toward another established vessel. Time lapse images were taken at an interval of every 60 seconds over a period of 2 hour and 11 minutes. The time scale is indicated in hh:mm:ss.
(MOV)

S6 Movie Csf1r-EGFP+ macrophages behave differently among more established, less dynamic vessels. Live imaging of *Csf1r-EGFP* ^*+/tg*^ *; Flk1-myr*::*mCherry* ^*+/tg*^ cornea implanted with PDGFBB/FGF2-releasing hydrogel at day 11 post-implantation. Csf1r-EGFP+ macrophages became less dynamic among more established vessels on the surface of implanted PDGFBB/FGF2 hydrogel at day 11 as compared to the subset that was interacting with the angiogenic front at day 4. Instead, these more static macrophages surrounded the vessels in a manner reminiscent of pericyte coverage suggesting a possible long term role as support cells to maintain vessel homeostasis. Time lapse images were taken at an interval of every 60 seconds over a period of 2 hour and 10 minutes. The time scale is indicated in hh:mm:ss.
(MOV)
