# Supplementary figures and images for: Improved Angiogenesis in Response to Localized Delivery of Macrophage-Recruiting Molecules
Source: PLoS One. 2015 Jul 1;10(7):e0131643. doi: 10.1371/journal.pone.0131643 (PMC4489184; doi:10.1371/journal.pone.0131643)

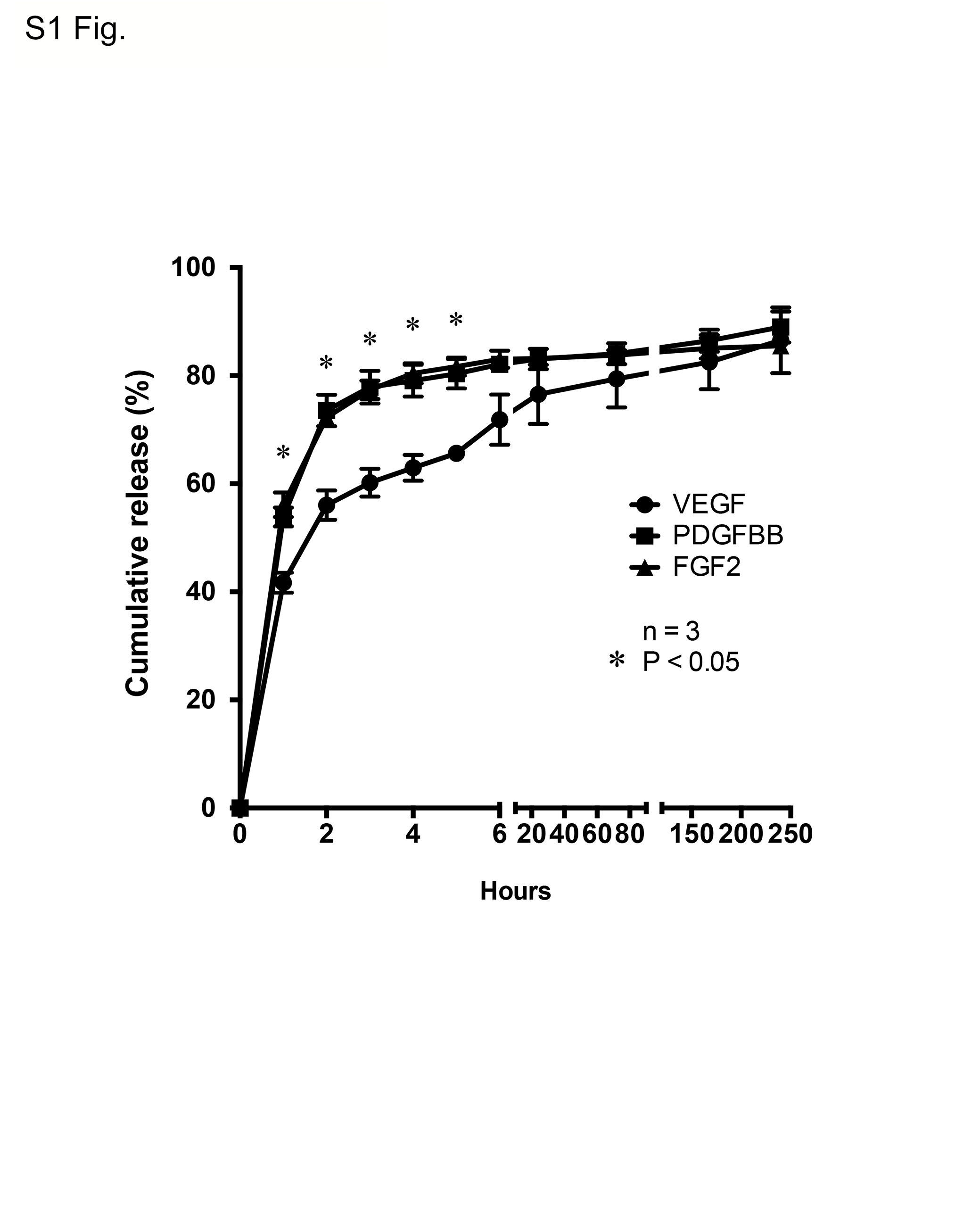

Supplement: S1 Fig — The release kinetics of PDGFBB, FGF2, and VEGF from PEGDA hydrogels (320 ng/gel) was tested in vitro. A fast release of the factors from the gels into PBS solution were observed within the first 6 hours, which followed by a slow release of the remaining factor over a period of 10 days. VEGF release was slower (n = 3, P<0.05) between hour 1 to 5 when compare to PDGFBB and FGF2. But by 6 hours and throughout the rest of the 10-day period, VEGF, FGF2, and PDGFBB have statistically indistinguishable release patterns. (TIF) [file pone.0131643.s001.tif]

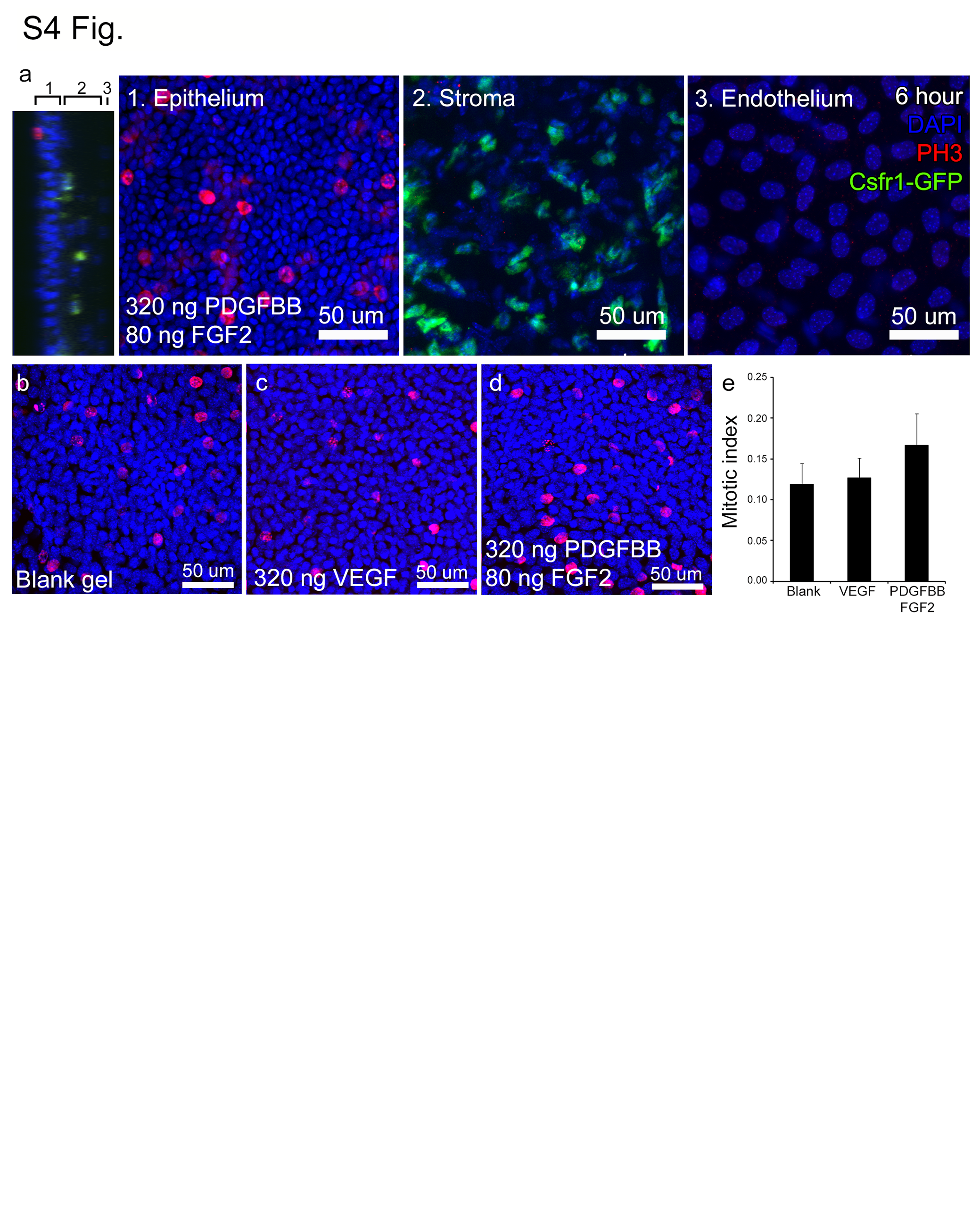

Supplement: S2 Fig — Z-stack image covering the full thickness of the cornea implanted with PDGFBB/FGF2 hydrogel at 6 hours post implantation shows that PH3+ nuclei are located exclusively in the epithelial layer of the cornea and Csf1r-EGFP+/PH3- cells reside within the corneal stroma (a). Corneas implanted with blank (b), VEGF- (c) and PDGFBB/FGF2-releasing hydrogels (d) were stained with PH3 at 6 hours post implantation. Quantification of the mitotic index showed no significant difference among all three groups (e). (TIF) [file pone.0131643.s002.tif]
